# Identification of a Novel Gnao-Mediated Alternate Olfactory Signaling Pathway in Murine OSNs

**DOI:** 10.3389/fncel.2016.00063

**Published:** 2016-03-29

**Authors:** Paul Scholz, Julia Mohrhardt, Fabian Jansen, Benjamin Kalbe, Claudia Haering, Katharina Klasen, Hanns Hatt, Sabrina Osterloh

**Affiliations:** Department of Cell Physiology, Ruhr-University BochumBochum, Germany

**Keywords:** olfactory pathways, GPCR signal transduction, electrophysiology, olfactory receptor neurons, patch-clamp techniques

## Abstract

It is generally agreed that in olfactory sensory neurons (OSNs), the binding of odorant molecules to their specific olfactory receptor (OR) triggers a cAMP-dependent signaling cascade, activating cyclic-nucleotide gated (CNG) channels. However, considerable controversy dating back more than 20 years has surrounded the question of whether alternate signaling plays a role in mammalian olfactory transduction. In this study, we demonstrate a specific alternate signaling pathway in Olfr73-expressing OSNs. Methylisoeugenol (MIEG) and at least one other known weak Olfr73 agonist (Raspberry Ketone) trigger a signaling cascade independent from the canonical pathway, leading to the depolarization of the cell. Interestingly, this pathway is mediated by Gnao activation, leading to Cl^−^ efflux; however, the activation of adenylyl cyclase III (ACIII), the recruitment of Ca^2+^ from extra-or intracellular stores, and phosphatidylinositol 3-kinase-dependent signaling (PI signaling) are not involved. Furthermore, we demonstrated that our newly identified pathway coexists with the canonical olfactory cAMP pathway in the same OSN and can be triggered by the same OR in a ligand-selective manner. We suggest that this pathway might reflect a mechanism for odor recognition predominantly used in early developmental stages before olfactory cAMP signaling is fully developed. Taken together, our findings support the existence of at least one odor-induced alternate signal transduction pathway in native OSNs mediated by Olfr73 in a ligand-selective manner.

## Introduction

The olfactory system is developed early during maturation in most mammals, indicating its crucial role in their daily survival. Odor detection is realized by a complex self-regenerating neuroepithelium, which is located in the nasal cavity and contains OSNs and supporting and basal cells (Mombaerts, 2004). OSNs in rodents collectively express ~1000 types of specific G protein-coupled receptors (GPCRs), called olfactory receptors (ORs), which are molecular sensors for incoming odor information (Buck and Axel, 1991). Olfactory reception starts with a conformational change of an OR elicited by its specific odorous ligands (Kato et al., 2008). Referring to the canonical signal transduction pathway, a heterotrimeric Gs protein (Golf) is activated, triggering the dissociation of the protein into an α and βγ subunit (Pace and Lancet, 1986; Jones and Reed, 1989; Belluscio et al., 1998). Gαolf activates ACIII, causing an increase in cAMP, which in turn opens Ca^2+^- and Na^+^-conducting cyclic nucleotide-gated (CNG) channels (Bakalyar and Reed, 1990; Dhallan et al., 1990). Thereby, OSNs are depolarized and Ca^2+^-activated Cl^−^ (CACL) channels are opened, leading to enhanced depolarization via Cl^−^ efflux (Kleene and Gesteland, 1991; Stephan et al., 2009; Rasche et al., 2010). However, considerable controversy dating back more than 20 years has surrounded the question of whether alternate signaling pathways could play a role in mammalian olfactory transduction (Breer et al., 1990). For OSNs, the PI-signaling pathway has been hypothesized as a possible alternate pathway (Ache, 2010). It was demonstrated in native OSNs that odorants can elevate levels of Phosphatidylinositol (3,4,5)-trisphosphate (PIP3), which in turn leads to an inhibition of the odor-evoked currents (Spehr et al., 2002; Brunert et al., 2010; Ukhanov et al., 2010, 2011). Furthermore, there are several studies indicating an influence of DAG- and PLC-dependent alternative olfactory signaling pathways in specific subsets of OSNs (Yu et al., 2014) and specialized OSNs expressing Trace amine–associated receptors (TAARs; Liberles and Buck, 2006) or Guanylyl cyclase-D (GC-D; Juilfs et al., 1997) instead of the ORs. Even more complexity in signaling modes is attained because heterologously expressed ORs can activate different G proteins in a stimulus-dependent manner, leading to a duality of intracellular signaling effects (Ukhanov et al., 2014). Generally, a second sensory pathway might promote the system to elicit, fine-tune, regulate, or diminish odor responses. Despite these further attempts to explore alternate signaling in OSNs, some molecular components, especially in the native system, are missing or poorly understood. Therefore, we aimed to find and characterize such an alternate signaling pathway in the native system, focusing on OSNs that express the well-characterized Olfr73 and its known ligands. By screening these ligands, we showed that the currents elicited by at least two known Olfr73 agonists (MIEG and RK) are mediated by an as-yet-unexplored signaling cascade. This alternate pathway is independent from olfactory canonical signaling in that the binding of MIEG to Olfr73 promotes the activation of Gnao, leading to Cl^−^ efflux and to the depolarization of the cell. In contrast, the strong agonist vanillin activated the known canonical olfactory pathway when applied to the same cells. Taken together, our study provides new insights into olfactory signaling by revealing an as-yet-unknown excitatory signaling pathway.

## Materials and methods

### Ethics statement

Animals were housed and treated in accordance with the European Union Community Council guidelines. All animal procedures were in compliance with the European Union legislation (Directive 86/609/EEC), FELASA (Federation of Laboratory Animal Science Associations) recommendations, were approved by the local animal use and care committee (The North Rhine-Westphalia State Environment Agency) and were conducted according to all applicable laws. All experiments were performed so as to minimize animal suffering.

### Animals

All experiments were performed using juvenile (p-2–p-5) or adult male (50%) and female (50%) transgenic *Olfr73* (Oka et al., 2006) mice that expressed GFP driven by the eugenol receptor promoter.

### Acute slice preparation

Transgenic *Olfr73* mice (postnatal day 2–5; Oka et al., 2006) were sacrificed by decapitation. The skin, lower jaw and incisors were removed before the head was dissected in ice-cold oxygenated extracellular solution. Afterwards, the mouse head was transferred into 4% (w/v) low-gelling-temperature agarose (Sigma-Aldrich, Munich, Germany) and acute coronal olfactory epithelium slices (300-μm) were prepared using a vibratome (VT1000S, Leica Microsystems, Wetzlar, Germany). Slices were collected in a Perspex chamber filled with cooled oxygenated extracellular solution until measurement. The extracellular solution contained 120 mM NaCl, 25 mM NaHCO3, 5 mM KCl, 1 mM CaCl2, 1 mM MgSO4, and 1 mM N,N-Bis-(2-hydroxyethyl)-2-aminoethane sulfonic acid (300 mOsm, pH 7.3).

### Electrophysiology

For electrophysiological characterization, acute slices of the OE were transferred to a recording chamber (Slice Mini Chamber, Luigs, and Neumann) and submerged in oxygenated extracellular solution. To prevent the acute slice from floating, a steel-wired slice-hold-down (SHD26H/15, Hugo Sachs Elektronik, Harvard Apparatus GmbH) was used. Single neurons were identified using a confocal microscope (Leica DM 6000 CFS, Leica Microsystems). Patch pipettes (6–9 MΩ) were pulled using borosilicate glass capillaries (GB150TF-8P, Science Products) processed by a PC-10 vertical two-step puller (Narishige Instruments) and fire-polished (MF-830 Microforge, Narishige Instruments). Patch clamp experiments were performed in the whole-cell configuration and recorded using an EPC-10 amplifier controlled by PatchMaster 2.20 (HEKA Elektronik, Lambrecht/Pfalz, Germany).

The pipette solution contained 143 mM KCl, 10 mM HEPES, 1 mM EGTA, 2 mM KOH, 1 mM MgATP, and 0.5 mM NaGTP (290 mOsm, pH 7.1). The liquid junction potential was calculated (JP-CalcW software) and automatically subtracted. The pipette and cell membrane capacitance were compensated during the experiment automatically. Experiments were performed by applying continuously oxygenated extracellular solution and using a holding potential of −56 mV. The recordings were filtered using Bessel filters at 10 kHz and at 2.9 kHz and the signals were sampled at 10 kHz. Odorants and inhibitors were diluted to the required concentration in Ringer's solution, and stored in separate tanks which were connected to the application system. The application was realized using a pressure-driven microcapillary system. A thin microcapillary, which generates a continuous stream of Ringer's solution, was placed next to the OE. During the experiments, computer-controlled odor and/or inhibitor stimuli from the different storage tanks were applied through this microcapillary. All slices were used for one measurement only, to prevent contamination.

### Quantitative real-time PCR

For real-time qPCR, we designed specific primer pairs to amplify defined regions (180–250 bp) within the examined genes. SYBR Green Mix (Bio-Rad) was used for amplification and samples were run on a Mastercycler realplex^2^ (Eppendorf). The primer pairs for each gene are listed in Supplementary Table 1.

### Co-immunoprecipitation and western blotting

OE lysate from BL6 mice (p-2–p-5) was used to perform co-immunoprecipitation experiments. The OE was minced in RIPA buffer [150 mM NaCl, 50 mM Tris-HCl, 1% Nonidet, 0.5% sodium deoxycholate (w/v), 0.1% SDS (w/v) and protease inhibitors] using the Catch & Release system (Catch & Release v2.0, Millipore). Samples were centrifuged for 10 min at 10,000 g, and the supernatant was isolated and kept on ice. The precipitate was minced in RIPA buffer and centrifuged again before both supernatants were pooled. The lysate (4 mg total protein) was incubated with primary antibodies (each 0.8 mg) overnight at 4°C in a spin column. After several washing steps, the proteins were eluted from the column using a denaturing elution buffer. Unspecific IgG (0.8 mg) was used for precipitation as control. Samples were loaded onto a SDS gel and immunoblotting was performed (Porablot NCL nitrocellulose membrane, Machery-Nagel). Interactions were detected using specific antibodies (1:50–1:250) and the enhanced chemiluminescence (ECL) detection system (GE Healthcare). The used antibodies are listed in Supplementary Table 1.

### Peptide microarrays

CelluSpotsTM Peptide Arrays (Intavis AG) were blocked for 2 h at room temperature with a 3% protein mixture (3K Eiweiss Shake, Layenberger) in NaCl/Tris/Tween. The arrays were incubated with heterologously expressed GnaO fused to HA at 4°C overnight. After several washing steps using NaCl/Tris/Tween, the microarrays were incubated with anti-HA antibodies (1:250) for 4 h at room temperature. Afterwards, HRP-coupled secondary antibodies and the ECL plus western blotting detection reagent (GE Healthcare) were used for visualization. Hits were considered positive when a signal was detectable in 3 out of 3 experiments.

### Data analysis

Electrophysiological data were analyzed off-line using IGOR Pro 6.05 (Wave Metrics) with Neuromatic 2.0 (Jason Rothman) and Excel 2010 (Microsoft) software.

Microarray data were obtained using VilberLourmat Fusion Software. Spot intensities were analyzed with TIGR Spotfinder 3.2.1. Intensities (0–255) were calculated as the mean values for spots in which a positive signal was observed in 3 out of 3 experiments. The individual highest intensity of one array was set as 100% and all other intensity values were normalized to this.

Statistical analyses were performed using an unpaired two-tailed Student's *t*-test as a built in function in Excel. *P*-values were assessed as follows: 0.05 < ^*^; 0.01 < ^**^; 0.005 < ^***^; ns, non-significant. Results are presented as the mean ± s.e.m. The number inside the columns represent the n numbers of the experimental approach.

### Solutions

**Physiological extracellular solution (oxygenated):** 120 mM NaCl, 25 mM NaHCO3, 5 mM KCl, 1 mM CaCl2, 1 mM MgSO4, 1 mM N,N-Bis-(2-hydroxyethyl)-2-aminoethane sulfonic acid (300 mOsm, pH 7.3)

**Physiological extracellular solution (non-oxygenated):** 145 mM NaCl, 5 mM KCl, 1 mM CaCl_2_, 1 mM MgCl_2_, 10 mM HEPES (300 mOsm, pH 7.3)

**Low Ca**^2+^ **solution (600 nM):** 135 mM NaCl, 5 mM EGTA, 3,45 mM CaCl_2_, 5 mM KCl, 10 Mm NaOH, 1 mM MgCl_2_, 10 mM HEPES (300 mOsm, pH 7.3)

**Low Ca**^2+^ **solution (600 nM)** + **NMDG:):** 5 mM EGTA, 3,45 mM CaCl_2_, 10 Mm NaOH, 1 mM MgCl_2_, 140 mM NMDG-Cl, 10 mM HEPES (300 mOsm, pH 7.3)

**Pipette solution:** 143 mM KCl, 10 mM HEPES, 1 mM EGTA, 2 mM KOH, 1 mM MgATP, 0.5 mM NaGTP (290 mOsm, pH 7.1)

## Results

### MIEG as a weak Olfr73 agonist

One major challenge regarding studies on single OSNs is the reproducibility of the results, as the murine OE encompasses ~1000 different types of OSNs, all expressing different types of ORs. Therefore, it is crucial to optically recognize neurons expressing the same OR to ensure the reliability of the obtained results. To solve this issue, we decided to perform our experiments on Olfr73-expressing OSNs, as we could use transgenic Olfr73-GFP mice (Oka et al., 2006), allowing us to identify Olfr73-positive OSNs for further experiments. Furthermore, Olfr73 is one of the best characterized murine ORs, with various known strong and weak agonists (Baud et al., 2011), and most of the previously performed alternate olfactory signaling studies analyzed this receptor (Oka et al., 2004; Ukhanov et al., 2014).

**Figure 1:**
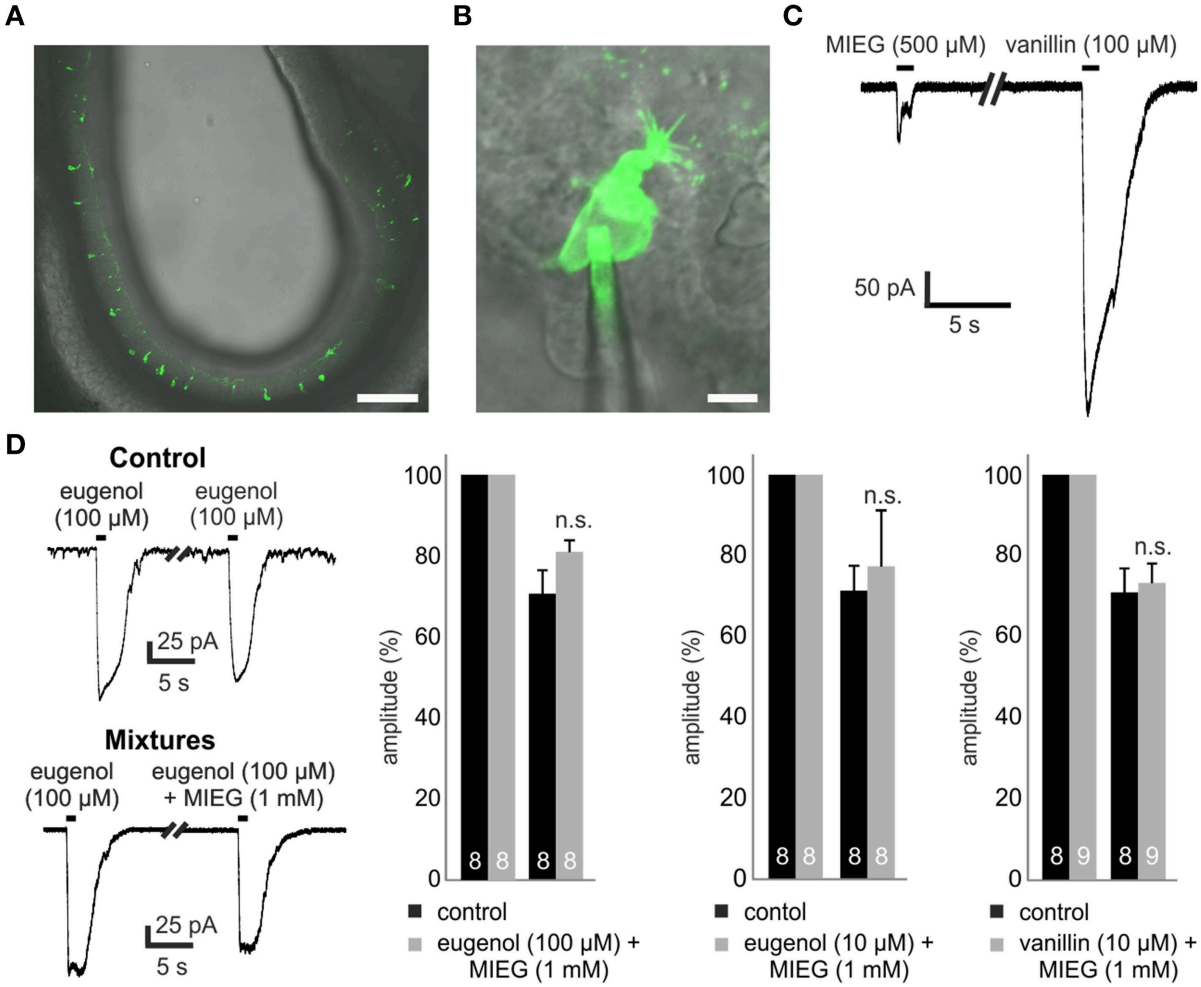
**Experimental procedure and mixture of agonists. (A)** 300-μm thick coronal acute slice through the OE of a transgenic Olfr73-GFP mouse. Scale bar, 100 μm **(B)** Identification of single GFP-labeled Olfr73 neurons for whole-cell patch clamp measurements. Scale bar, 5 μm **(C)** Representative patch clamp trace comparing the odor-induced receptor currents elicited by a 1 s application of MIEG (500 μM) or vanillin (100 μM), respectively. **(D)** Mixtures of strong agonists (vanillin, eugenol) (100/10 μM) with MIEG (500 μM) to evaluate MIEGs' antagonistic potential. Mixtures were applied for 1 s every 30 s and the amplitudes were compared to control measurements (vanillin and eugenol applied separately).

To perform patch-clamp experiments, we prepared 300 μm-thick acute slices of the main olfactory epithelium obtained from p2 to p5 Olfr73-GFP mice (Figure 1A). We utilized a combined confocal laser-scanning/patch-clamp setup to visualize GFP-fluorescent OSNs and to perform whole-cell patch-clamp experiments with these neurons (Figure 1B).

We first tested MIEG, which is described as a weak agonist of Olfr73 (Baud et al., 2011) with a dose-dependent inhibitory effect on the currents elicited by strong agonists (Oka et al., 2004). To study the receptor currents generated by MIEG (500 μM), we applied MIEG for 1 s, compared the elicited currents to those generated by the strong agonist vanillin (100 μM), and detected significant differences in their peak maximal amplitudes (vanillin = 481.8 ± 56.8 pA, MIEG = 104.6 ± 49.5 pA; Figure 1C). To exclude a non-OR-derived activation, we performed experiments where we applied MIEG on several random (non-GFP-labeled) OSN and could not detect any responses using our patch clamp setup (data not shown).

### Antagonistic effect of MIEG

In previous Ca^2+^ imaging experiments, it was shown that MIEG can influence/inhibit the elicited Ca^2+^ response by the strong Olfr73 agonist eugenol in a dose-dependent manner (Oka et al., 2004). To further investigate this inhibitory effect, we performed whole-cell patch-clamp experiments mixing MIEG (1 mM) with the known strong agonist eugenol (100 μM) as described in the above-mentioned paper. After an initial 1-s application of the strong agonist alone, the mixture was applied for 1 s and the elicited currents were normalized to the initial stimulus and compared to the control experiments. We demonstrated that the eugenol-induced receptor currents showed no significant changes in their maximal amplitudes [eugenol (100 μM) = 70.8 ± 5.8%, mixture = 81.4 ± 2.8%] when coapplied with MIEG in the native system (Figure 1D). To further investigate this antagonistic effect described by Oka et al. (2004), we repeated the experiments but lowered the concentration of our strong agonist eugenol to a sub-maximal concentration (10 μM; Baud et al., 2011). Furthermore, we tested a second known strong agonist (vanillin 10 μM) but could not detect significant changes in the main maximal amplitudes in both experimental approaches [eugenol (10 μM) = 71.4 ± 6.1%, mixture = 77.3 ± 13.8%] [vanillin (10 μM) = 70.9 ± 5.9%, mixture = 73.2 ± 4.7%; Figure 1D].

### Inhibition of canonical signaling proteins

**Figure 2:**
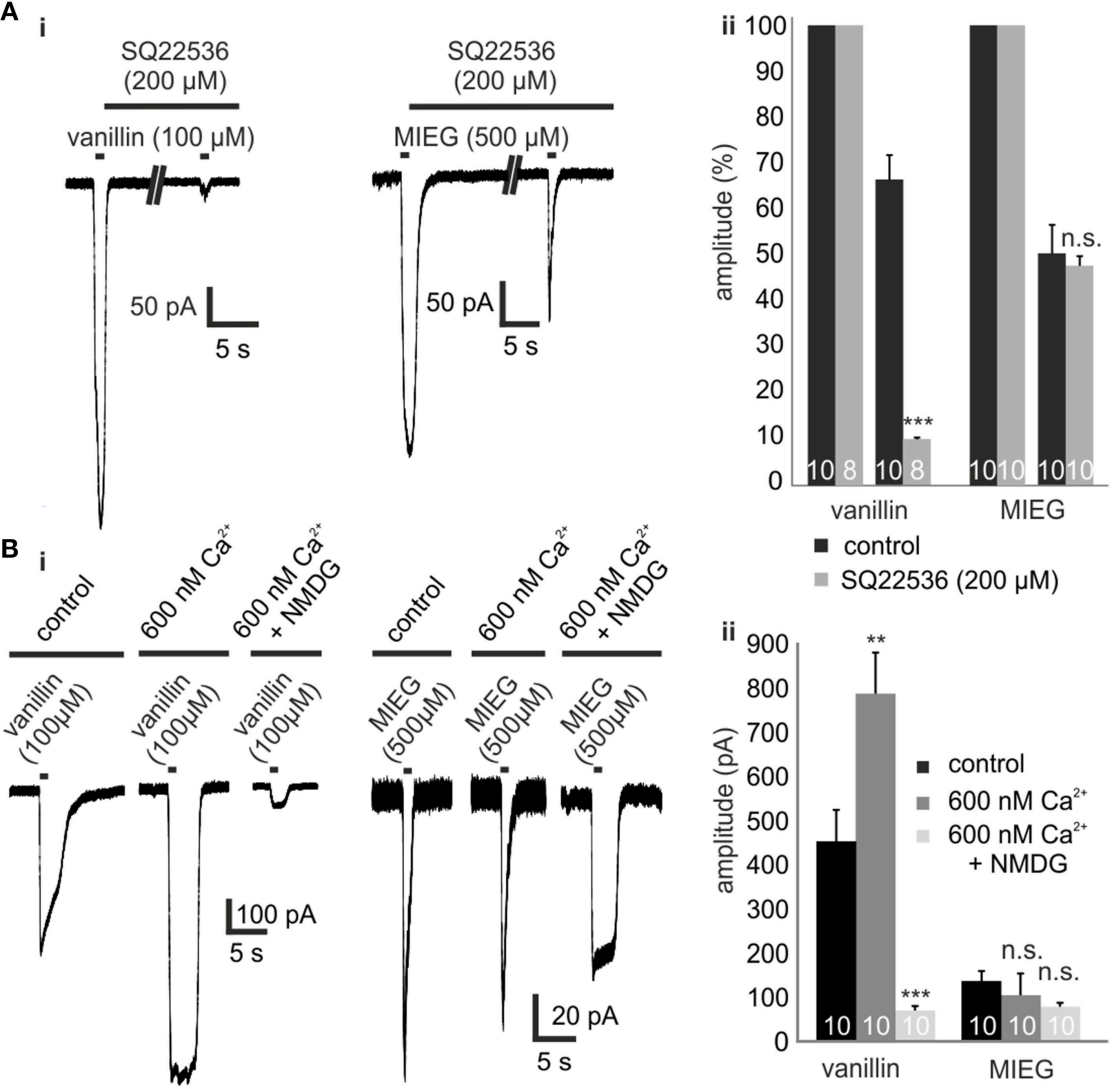
**Inhibition of olfactory cAMP signaling proteins ACIII and CNGC. (Ai)** Representative traces showing the odor-induced currents elicited by 1-s application of vanillin (100 μM) or MIEG (500 μm) after 5 min of preincubation with SQ22536 (200 μM) in comparison to the control response prior to SQ22536. **(Aii)** Quantification of the receptor current amplitudes collected from the AC3 blocking experiments. **(Bi)** Exemplary traces monitoring the odor-induced receptor currents elicited by vanillin (100 μM)/MIEG (500 μM) using different extracellular conditions (control, 600 nM Ca^2+^, 600 nM Ca^2+^ + NMDG). **(Bii)** Bar graphs illustrating the mean maximal amplitudes under these different conditions.

To test whether the MIEG-induced currents rely on an alternate pathway and are possibly mediated independent of olfactory cAMP signaling, we pharmacologically blocked different components involved in this pathway and analyzed the elicited currents. The first key element essential for canonical olfactory signaling is ACIII, which is activated by Golf. To test whether the odor-induced currents generated by MIEG are based on ACIII activation, we used the ACIII inhibitor SQ22536. After an initial 1-s application of MIEG (500 μM) or vanillin (100 μM), we pre-incubated the acute slices with SQ22536 (200 μM) for 5 min, applied MIEG/vanillin for 1 s again (Figure 2Ai), normalized the odor-induced amplitudes to the initial response, and compared the results to control experiments without the inhibitor. MIEG-evoked currents showed no significant changes (46.5 ± 8.0%) in their amplitude compared to the control measurements (51.0 ± 6.2%), whereas vanillin-induced currents were significantly reduced (control = 67.2 ± 5.3%, SQ22536 = 10.2 ± 0.3%) after the inhibition of ACIII (Figure 2Aii). These results indicate the independence of MIEG signaling from canonical olfactory signaling. We next investigated the involvement of CNG channels, which are the primary ion channels involved in canonical signaling and are activated by cAMP generated by ACIII (Bakalyar and Reed, 1990; Dhallan et al., 1990). When activated, Ca^2+^ and Na^+^ can pass through the channel into the cell, leading to a depolarization. To examine the involvement of CNG channels in MIEG signaling, we first conducted experiments using low (600 nM) extracellular Ca^2+^ conditions (Material and Methods). Therefore, we applied our agonists for 1 s under low extracellular Ca^2+^ conditions and compared the elicited amplitudes with those detected under physiological conditions. Interestingly, the MIEG-induced currents were unaffected (control = 104.6 ± 49.5 pA, low Ca^2+^ = 96.1 ± 18.0 pA) by these conditions, whereas vanillin-elicited currents were significantly increased (control = 481.8 ± 56.8 pA, low Ca^2+^ = 787.1 ± 92.4 pA; Figures 2Bi,ii). To further strengthen these results, we additionally lowered the extracellular Na^+^ and K^+^ concentration to 10 mM and 0 mM, respectively, by replacing NaCl and KCl with NMDG-CL. NMDG-Cl has similar chemical properties to Na^+^ and K^+^, but CNG channels are impermeable to NMDG ions (Simasko, 1994; Chidekel et al., 1997; Rose and Ransom, 1997). Using the same experimental procedure as described for low Ca^2+^ measurements, we could show that the vanillin-induced currents were significantly decreased (low Ca^2+^ = 787.1 ± 92.4 pA, low Ca^2+^ + NMDG = 69.7 ± 11.0 pA) due to the lack of Na^+^ ions. In contrast to this, the MIEG-evoked currents were not significantly affected (low Ca^2+^ = 96.1 ± 18.0 pA, low Ca^2+^ + NMDG = 65.8 ± 6 pA; Figures 2Bi,ii).

**Figure 3:**
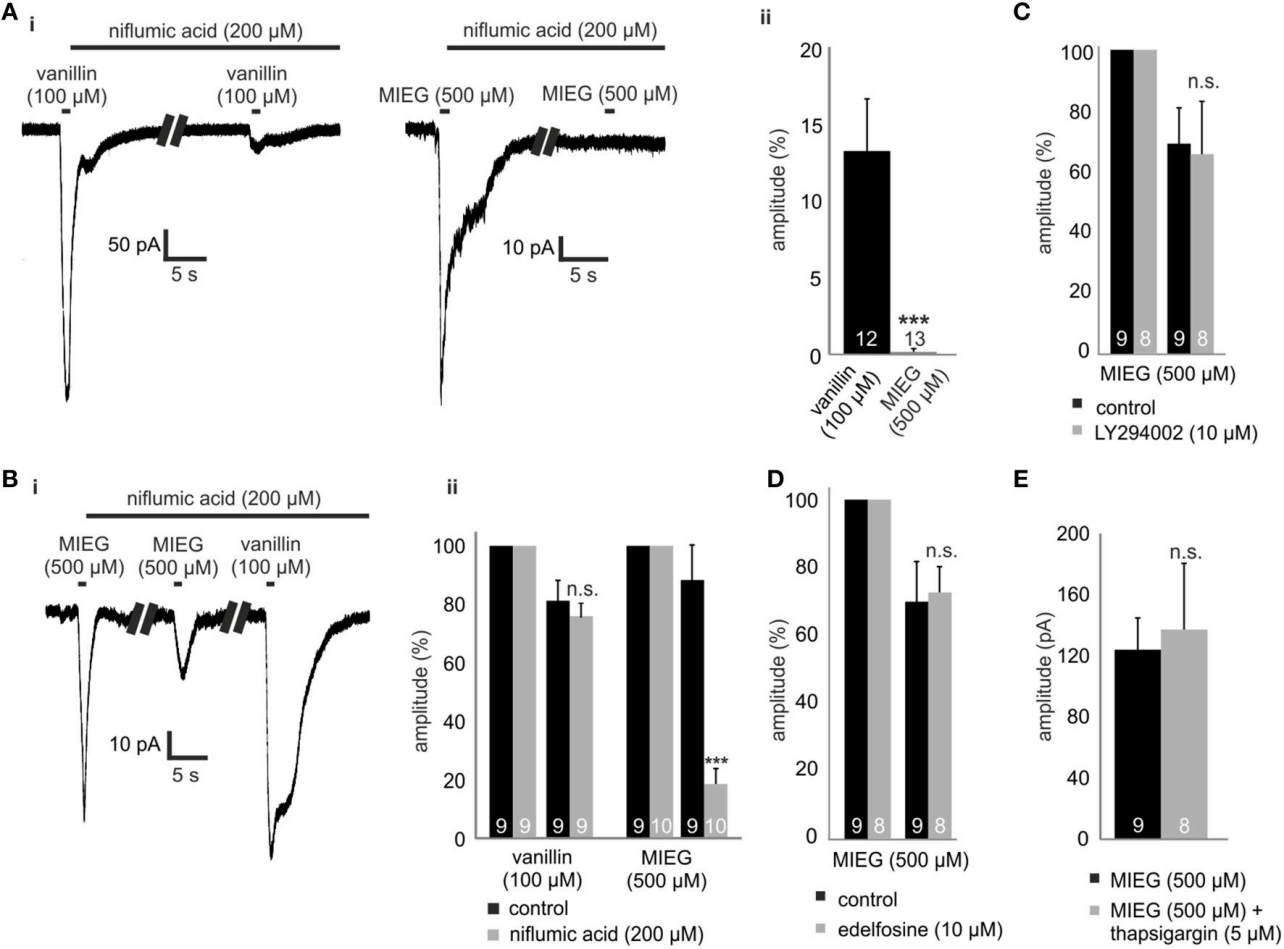
**Inhibition of CACLC, PI signaling proteins and Ca^2+^ from intracellular stores. (Ai)** Patch clamp recordings monitoring the odor-elicited currents generated by vanillin (100 μM)/MIEG (500 μM) after CACLS inhibition. 200 μM of niflumic acid, preincubated for 1 min, was used for CACL inhibition and odor responses were compared to the first applied control stimulus. **(Aii)** Bar diagram displaying the amplitudes generated by vanillin/MIEG after CACLC inhibition normalized to a control response (100%). **(Bi)** Exemplary traces showing receptor currents using low Ca^2+^ + NMDG extracellular ringer solution. Additionally, niflumic acid was incubated after the first MIEG control application to inhibit CACLC. **(Bii)** Bar diagrams showing the low Ca^2+^, NMDG, and niflumic acid results for vanillin and MIEG normalized to the first control application and compared to control experiments. **(C)** Evaluation of the receptor currents after 1 min LY (10 μM) preincubation for MIEG (500 μM). Amplitudes were normalized to the first control application and compared to the control experiments. **(D)** Evaluation of the receptor currents after 1 min edelfosine (10 μM) preincubation for MIEG (500 μM). Amplitudes were normalized to the first control application and compared to the control experiments. **(E)** Bar diagram displaying amplitudes of the MIEG-elicited currents after the Ca^2+^ depletion from intracellular stores using 12 min thapsigargin (5 μM) preincubation. Amplitudes were normalized to the first control application and compared to control experiments.

The final step in canonical olfactory signaling is the activation of CACLC, magnifying the depolarization of the cell (80% of the receptor currents; Reisert et al., 2003). To investigate the potential involvement of CACLC in MIEG signaling, we inhibited these channels using an unspecific blocker for chloride channels (niflumic acid 200 μM; Knauf and Mann, 1984). We pre-incubated niflumic acid after an initial 1-s agonist stimulus for 1 min. Afterwards, we applied our agonist for 1 s again (Figure 3Ai), evaluated the elicited responses, normalized them to the initial response, and compared the results to control experiments. We observed that the MIEG-induced currents were completely abolished, whereas the vanillin-induced currents where significantly decreased to 13.5 ± 3.4% (Figure 3Aii). Additionally, we combined the previously described low Ca^2+^ and NMDG approach and additionally blocked Cl^−^-channels using niflumic acid. The MIEG-induced currents were significantly decreased (control = 88.0 ± 11.9%, niflumic acid = 18.5 ± 5.1%), whereas the vanillin-induced currents showed no significant effect (control = 80.95 ± 7.0%, niflumic acid = 75.7 ± 4.4%; Figures 3Bi,ii).

### Pi signaling and Ca^2+^ from intracellular stores

PI signaling has been proposed as an possible alternate olfactory pathway in recent decades (Breer et al., 1990; Spehr et al., 2002; Ache, 2010; Brunert et al., 2010; Klasen et al., 2010; Ukhanov et al., 2010). To determine whether this signaling cascade could be the mediator of MIEG signaling, we inhibited PI3K and PLC, respectively, using the blockers LY294002 (Vlahos et al., 1994) and edelfosine (Powis et al., 1992). After an initial 1-s application of the agonist, we pre-incubated the acute OE slices for 1 min with LY294002 or edelfosine and afterwards applied the agonist again for 1 s. The elicited currents were then normalized to the initial application and compared to control measurements without the inhibitors. We showed that the MIEG pathway is independent from PI signaling, as we could not detect any changes in the odor-induced currents in the presence of the blockers (control = 70.5 ± 11.3%, LY294002 = 67.1 ± 17.8%, edelfosine = 72.4 ± 7.4%; Figures 3C,D). To exclude the possibility that Cl^−^ channels could be activated through Ca^2+^ release from intracellular stores, we depleted them by 15 min of pre-incubation with 10 μM thapsigargin. Afterwards, we applied MIEG for 1 s and compared the elicited currents to those generated in control experiments without thapsigargin. Our results showed that the pre-incubation with thapsigargin had no effect on the MIEG-generated currents, as they showed no changes in their amplitudes (control = 124.2 ± 20.7 pA, thapsigargin = 113.0 ± 17.7 pA; Figure 3E).

### Raspberry ketone

**Figure 4:**
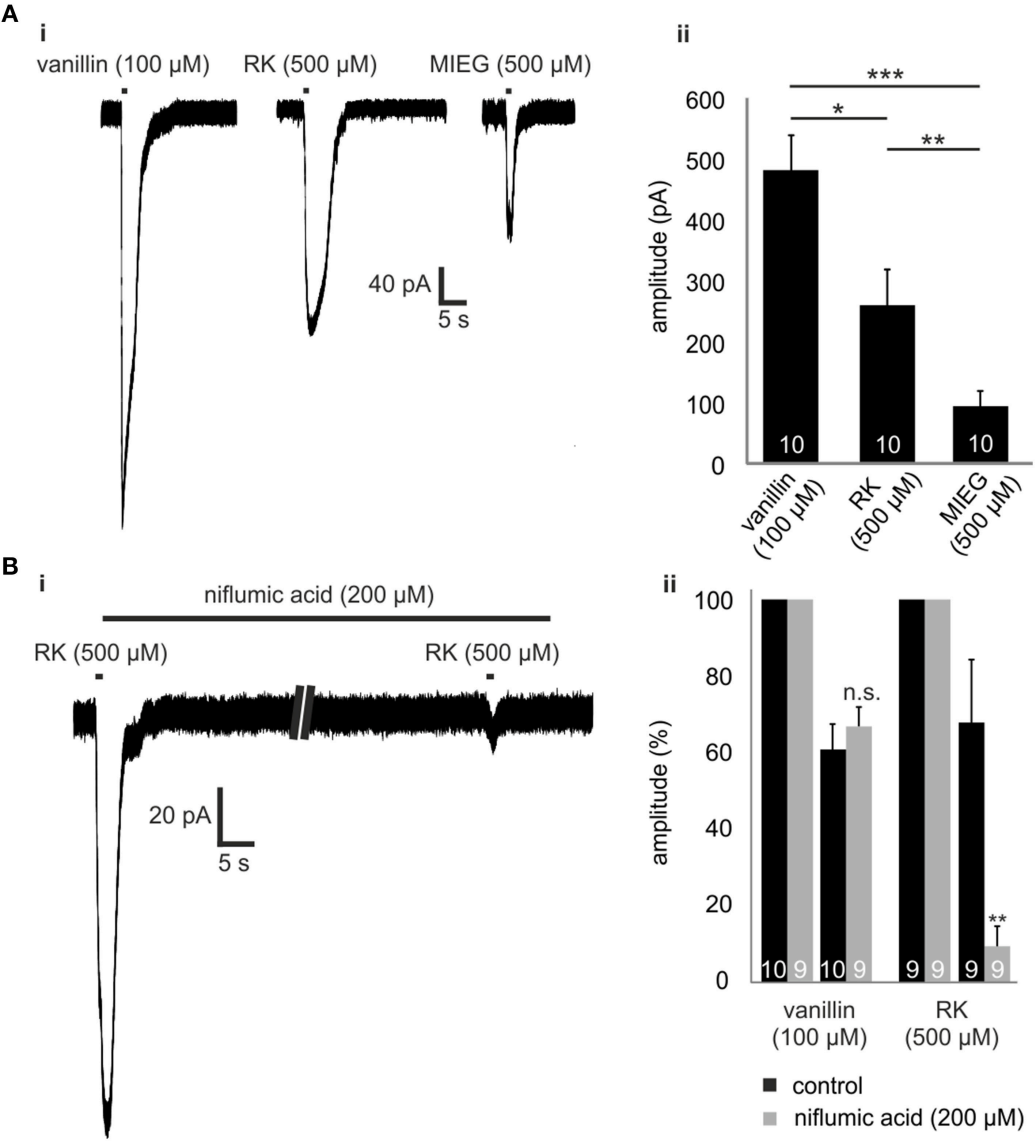
**Raspberry Ketone**. **(Ai)** Comparison of the odor-elicited currents of vanillin (100 μM), MIEG (500 μM), and Raspberry Ketone (500 μM). **(Aii)** Bar diagram showing the mean amplitudes elicited by these odors when applied for 1 s. **(Bi)** Patch clamp experiments using low extracellular Ca^2+^ + NMDG conditions. After the primary application of RK, the sample was incubated for 1 min with niflumic acid before RK was applied again. **(Bii)** Amplitudes of the elicited currents were normalized to the first control signal and compared to control measurements without niflumic acid incubation.

Raspberry ketone (RK) is one of the weakest agonists (based on cAMP accumulation) for Olf73 activation (Baud et al., 2011). To test whether RK-mediated signaling is also promoted by this alternate MIEG signaling pathway, we first applied RK (500 μM) for 1 s and compared the odor-induced currents to signals elicited by vanillin (100 μM) and MIEG (500 μM; Figure 4Ai). Using the same odor concentration, we showed that amplitudes elicited by RK (257.7 ± 56.6 pA) were stronger than MIEG-elicited currents (104.6 ± 49.5 pA) but significantly smaller than vanillin-induced currents (481.8 ± 56.8 pA; Figure 4Aii). Furthermore, we performed the low Ca^2+^, NMDG and niflumic acid measurements as previously described using RK. We showed that RK-induced signaling was also mediated by the alternate MIEG pathway, as the RK-elicited currents showed a significant decrease in their amplitudes (control = 68.4 ± 16.6 pA, niflumic acid = 9.5 ± 5.3 pA), whereas the vanillin-elicited signals were unaffected (control = 61.3 ± 6.8 pA, niflumic acid = 67.4 ± 5.1 pA; Figures 4Bi,ii).

### Gnao as the mediator of MIEG signaling

**Figure 5:**
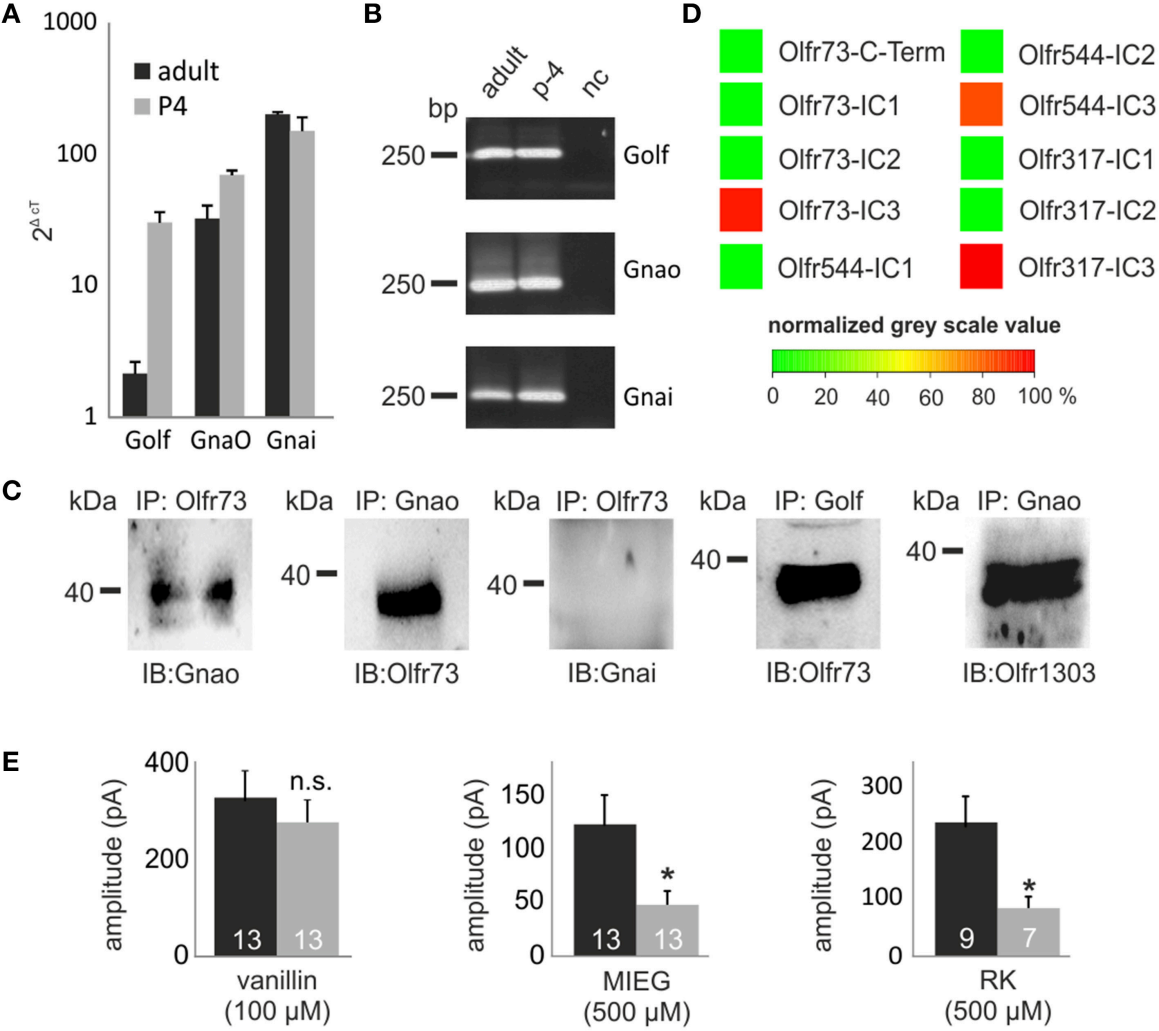
**GO involvement**. **(A)** Quantitative real-time PCR experiments showing the expression rate of the three G Protein alpha subunits (G_olf_ G_nao_ G_nai_) in the olfactory epithelium. **(B)** The-qPCR products were plotted on a gel to proof the specificity of the used primers and to rule out false positive results due to gDNA contamination. **(C)** Co-immunoprecipitations showing the capability of Gnao, Golf and Gnai to interact with Olfr73. Additionally, the interaction between one randomly chosen OR (Olfr1303) and Gnao are displayed. **(D)** Peptide microarrays spotted with potential OR binding sites and incubated with heterologously expressed Gnao. **(E)** Patch clamp measurements using pertussis toxin (500 μM) to inhibit Gnao activity. Pertussis toxin was preincubated for 2 h. Afterwards, vanillin, MIEG or RK were applied and the odor-induced amplitudes were evaluated and compared to control experiments. Nc, negative control; Δct, ct value of a housekeeping gene (beta actin)—ct value of the evaluated gene.

Previously, we identified strongly expressed genes in single Olfr73-expressing OSNs that could play a role in alternate signaling. Analyzing these next generation sequencing (NGS) results, we identified Gnao (141.4 FPKM) as the second highest expressed G protein alpha subunit beside Golf (393.3 FPKM) in Olfr73-positive neurons (Scholz et al., 2016). To validate these data, we performed quantitative real-time PCR analysis of cDNA generated from OE of adult and P4 mice using specific primers for the three highest expressed G protein alpha subunits detected in the RNA-Seq experiments (Golf, Gnao, Gnai). These experiments confirmed that Gnao is the second highest expressed G protein alpha subunit in adult mice (Golf = 2.2 ± 0.5 2^ΔcT^, Gnao = 32.7 ± 8.2 2^ΔcT^, Gnai = 202.8 ± 7.3 2^ΔcT^), whereas Golf (30.5 ± 6.0 2^ΔcT^) and Gna_o_ (70.1 ± 5.4 2^ΔcT^) showed similar expression rates in p-4 mice (Figures 5A,B).

To identify putative interaction partners of the highly abundant Gnao protein and to unravel its binding specifications, we performed co-immunoprecipitation experiments using specific antibodies against Olfr73 and Gnao. Pull-down experiments using lysate of the adult murine olfactory epithelium demonstrated that Gnao interacts with Olfr73 in the native system (Figure 5C). To validate this data, we performed additional co-immunoprecipitation experiments in which we could demonstrate an interaction between Olfr73 and Golf, whereas Gnai did not interact with Olfr73 (Figure 5C). Furthermore, we tested the capability of Gnao to interact with another, randomly chosen OR (Olfr1303) and could show its interaction capability with Gnao (Figure 5C). To further characterize this binding, we designed peptide microarrays encompassing the potential binding sites of different ORs (C-terminus and intracellular loops Ritter and Hall, 2009). We incubated these microarrays using heterologously expressed and purified Gnao proteins and monitored their relative binding affinities. In contrast to the C-Termini (Supplementary Figure 1), we detected high relative binding affinities for Gnao to the third intracellular loop of Olfr73 and the other observed ORs (Figure 5D). As Gnao is highly expressed in Olfr73-positive neurons and is capable of interacting with Olfr73, it could be a promising candidate for triggering the MIEG-induced signals. To investigate this, we conducted experiments using the Gnao/i inhibitor pertussis toxin (Burns, 1988). We pre-incubated the acute slices for 3 h with pertussis toxin (500 μM) and afterwards applied our agonists (vanillin 100 μM, MIEG 500 μM, RK 400 μM) for 1 s. The detected signals were compared to control experiments without pertussis toxin. Afterward, we detected that the MIEG-and RK-elicited currents were significantly decreased (MIEG; control = 144.9 ± 32.3 pA, pertussis toxin = 56.1 ± 14.8 pA) (RK; control = 241.1 ± 47.5 pA, pertussis toxin = 87.9 ± 21.2 pA) in contrast to the vanillin-induced currents, which were not significantly altered (control = 326.7 ± 55.0 pA pertussis toxin = 266.2 ± 45.6 pA; Figure 5E).

## Discussion

In the present study, we characterized a previously unknown excitatory alternate OR-mediated signaling pathway in native murine OSNs, which is activated by Olfr73. Furthermore, this pathway coexists with the known canonical cAMP-mediated olfactory signaling pathway in the same Olfr73-expressing neurons, and both can be activated independently by the same OR in an odor-dependent manner. However, it is mediated by Gna_O_ activation followed by Cl^−^ efflux, leading to the depolarization of the neuron.

The concept of alternate signaling mediated by GPCRs was originally introduced in the 1990s. It was described that GPCRs can trigger a hierarchy of downstream signaling components, mediated primarily by the conformational change of the ligand-GPCR complex. Thereby, different ligands reproducibly shift the balance of the cell's signaling network toward different transduction pathways (Gurwitz et al., 1994; Kenakin, 1995) and is most commonly referred to as ligand-induced selective signaling or biased agonism (González-Maeso and Sealfon, 2012; Zocher et al., 2012).

To characterize such an alternate pathway in OSNs, we conducted electrophysiological and biochemical experiments using transgenic Olfr73-GFP mice and mainly focused on the known Olfr73 ligands vanillin and MIEG. MIEG seemed to be an interesting candidate, as its properties are controversial. First, MIEG was described as a potential antagonist which inhibits the odor-elicited currents generated by eugenol in a dose-dependent manner until the responses are completely abolished (Oka et al., 2004). However, a more recently published study identified MIEGs as an activator of Olfr73 (Baud et al., 2011). We first tried to reproduce these results from Oka et al. using our whole-cell patch clamp approach but could not validate the described inhibitory effect. Instead, we observed detectable responses generated by MIEG alone, validating it as an Olfr73 agonist. Based on these findings, we studied the possible involvement of MIEG in alternate signaling. Therefore, we conducted whole-cell patch clamp experiments in which we inhibited the proteins involved in canonical signaling and studied the odor-evoked currents elicited by MIEG. First, we analyzed the involvement of the ACIII, which is essential for cAMP signaling and activated by Golf. We showed that the MIEG-elicited currents did not depend on ACIII activation, indicating its independency from the canonical olfactory signaling pathway. Furthermore, we validated these findings, as we conducted several studies focusing on the involvement of CNG and CACL channels. Here, we analyzed the function of the CNG channels by lowering the extracellular Ca^2+^ concentration to interrupt the Ca^2+^ influx through these channels. Using these extracellular concentrations interrupting the canonical olfactory pathway, we first applied vanillin and detected increased currents which can be explained by the strong correlation between low extracellular Ca^2+^ and an increasing CNG channel conductibility for Na^+^ (Frings et al., 1992) leading to a strong Na^+^ influx under low Ca^2+^ conditions. To inhibit this Na^+^ influx, we replaced most of the Na^+^ by NMDG, which has similar chemical properties as Na^+^ but is unable to pass CNG channels (Simasko, 1994; Chidekel et al., 1997; Rose and Ransom, 1997). Because Na^+^ and Ca^2+^ influx are minimized under these conditions, the vanillin-induced currents were significantly decreased in contrast to the MIEG-elicited currents which were not significantly altered by these changes (low Ca^2+^, low Ca^2+^ + NMDG). In both previous experiments, we were not able to completely replace those ions (low Ca^2+^ = 600 nM/low Na^+^ = 10 mM) due to limitations regarding the robustness of the OSNs. By changing the ion concentrations of the extracellular solutions to more non-physiological surroundings, the membranes of the OSNs became more sensitive to mechanical stimuli. However, we believe that our performed experiments demonstrate the independence of the MIEG-elicited currents from canonical olfactory signaling using several different approaches. Furthermore, these low Ca^2+^ + NMDG conditions are appropriate to study the differences in both pathways in more detail. Under these conditions, the vanillin-induced currents exclusively depend on Na^+^ influx through the activation of CNG channels, as further processing is interrupted due to the inhibited Ca^2+^ influx. In contrast to this, our hypothesis is that the MIEG-induced currents are exclusively based on a Cl^−^ efflux through CACLC, allowing us to analyze them completely independent from each other. The generated currents of both agonists exhibited similar amplitudes under these conditions (low Ca^2+^ + NMDG), allowing more precise studies and a better comparability of the results.

Furthermore, we conducted experiments focusing on essential proteins involved in PI signaling and Ca^2+^ release from intracellular stores, as these pathways were discussed to promote alternate olfactory signaling. Thereby, we could show that our newly identified MIEG pathway is independent from the above-mentioned components (PI3K, PLC, Ca^2+^ from intracellular stores). Finally, we used these experimental approaches to identify another already described Olfr73 agonist (raspberry ketone) which activated the newly discovered MIEG signaling pathway as well.

Taken together, our patch clamp results showed that MIEG-evoked currents are generated independently from cAMP (canonical olfactory signaling pathway)/PI signaling and are exclusively based on Cl^−^ efflux, leading to the depolarization of the neuron. MIEG and cAMP signaling are both mediated by Olfr73, showing that Olf73 in native OSNs is able to trigger at least two different signaling pathways in a stimulus-dependent manner in one OSN.

To further characterize the newly identified pathway in more detail, we looked up our previously conducted single OSN Next Generation Sequencing results and detected high expression levels of *Gna*_*O*_ in juvenile (200 FPKM) and adult mice (133 FPKM; Scholz et al., 2016). We validated these findings by performing quantitative PCRs and further analyzed the role of Gnao. We conducted biochemical experiments and showed the capability of Gnao to interact with Olfr73 and the other tested ORs by binding to intracellular loop 3. Further patch clamp experiments in which the GnaO activity was inhibited by a pharmacological blocker revealed its essential role in MIEG signaling. Based on these results obtained by RNA-Seq, qPCR, microarrays and electrophysiology, we believe that Gnao is the most promising candidate for mediation of the MIEG pathway. Furthermore, we believe that Olfr73 is able to bind two distinct G-proteins (Golf/Go) on different interaction sites [C-Terminus (Katada et al., 2005)/3. intracellular loop] and is capable to activate them independently from each other in a ligand-dependent manner. These results also support a recent published paper in which the authors showed the capability of MIEG and other known agonists to activate heterologously overexpressed Golf/Gna15 in HEK293T cells in a ligand-dependent manner (Ukhanov et al., 2014). As Gnao is highly expressed in juvenile mice (before Golf and other cAMP signaling components are upregulated during maturation), this pathway might reflect an odor recognition system predominantly used in embryonic and juvenile mice before cAMP signaling is fully developed. Admittedly, less is known about the role and function of Gnao, even though it is the most abundant G protein α-subunit in neurons (Offermanns, 2003). Focusing on chemical senses, Gnao is known to be essential for the survival of primary accessory olfactory neurons (Tanaka et al., 1999). Additionally, it is the primary G protein alpha subunit that mediates the detection of peptide and protein pheromones in the vomeronasal organ (Chamero et al., 2011). Furthermore, Gnai/o family α-subunits, such as gustducin (Gnagust; Wong et al., 1996) or transducin (Gnat-r; Gnat-c; Calvert et al., 2000) are involved in sensory functions. Nevertheless, the linkage between Gnao and CACL channel activation is still unclear and further studies addressing this subject should clarify these missing steps.

Here, we elucidated an alternate signaling pathway in native Olfr73-positive murine olfactory sensory neurons. In contrast to the former published findings, this signaling pathway is independent from PI signaling and does not influence the canonical pathway mediated by the same receptor in these neurons. Furthermore, this alternate pathway is mediated by GnaO, which, in turn, opens CACL channels, leading to the depolarization of the cell.

## Author contributions

PS, SO, KK, and HH designed the study and interpreted the results. PS, SO, JM, BK, FJ, and CH performed the experiments and evaluated the data. PS and SO drafted the manuscript. All authors contributed to the final version of the manuscript.

### Conflict of interest statement

The authors declare that the research was conducted in the absence of any commercial or financial relationships that could be construed as a potential conflict of interest.

## Supplementary material

Supplementary Table 1 **Primers and Antibodies**.

Supplementary Figure 1 **Peptide microarrays spotted with 304 OR c-termini and incubated with heterologously expressed Gnao**.

## References

[B1] Ache B. W. (2010). Odorant-specific modes of signaling in mammalian olfaction. Chem. Senses 35, 533–539. 10.1093/chemse/bjq045 20519266 PMC2924424

[B2] Bakalyar H. Reed R. (1990). Identification of a specialized adenylyl cyclase that may mediate odorant detection. Science 250, 1403–1406. 10.1126/science.2255909 2255909

[B3] Baud O. Etter S. Spreafico M. Bordoli L. Schwede T. Vogel H.. (2011). The mouse eugenol odorant receptor: structural and functional plasticity of a broadly tuned odorant binding pocket. Biochemistry 50, 843–853. 10.1021/bi1017396 21142015

[B4] Belluscio L. Gold G. H. Nemes A. Axel R. (1998). Mice deficient in G(olf) are anosmic. Neuron 20, 69–81. 10.1016/S0896-6273(00)80435-3 9459443

[B5] Breer H. Boekhoff I. Tareilus E. (1990). Rapid kinetics of second messenger formation in olfactory transduction. Nature 345, 65–68. 10.1038/345065a0 2158631

[B6] Brunert D. Klasen K. Corey E. A. Ache B. W. (2010). PI3Kgamma-dependent signaling in mouse olfactory receptor neurons. Chem. Senses 35, 301–308. 10.1093/chemse/bjq020 20190008 PMC2854420

[B7] Buck L. Axel R. (1991). A novel multigene family may encode odorant receptors: a molecular basis for odor recognition. Cell 65, 175–187. 10.1016/0092-8674(91)90418-X 1840504

[B8] Burns D. L. (1988). Subunit structure and enzymic activity of pertussis toxin. Microbiol. Sci. 5, 285–287. 2908558

[B9] Calvert P. D. Krasnoperova N. V. Lyubarsky A. L. Isayama T. Nicoló M. Kosaras B.. (2000). Phototransduction in transgenic mice after targeted deletion of the rod transducin α-subunit. Proc. Natl. Acad. Sci. U.S.A. 97, 13913–13918. 10.1073/pnas.250478897 11095744 PMC17675

[B10] Chamero P. Katsoulidou V. Hendrix P. Bufe B. Roberts R. Matsunami H.. (2011). G protein Gαo is essential for vomeronasal function and aggressive behavior in mice. Proc. Natl. Acad. Sci. U.S.A. 108, 12898–12903. 10.1073/pnas.1107770108 21768373 PMC3150917

[B11] Chidekel A. S. Friedman J. E. Haddad G. G. (1997). Anoxia-induced neuronal injury: role of Na+ entry and Na+-dependent transport. Exp. Neurol. 146, 403–413. 10.1006/exnr.1997.6544 9270051

[B12] Dhallan R. S. Yau K. W. Schrader K. A. Reed R. R. (1990). Primary structure and functional expression of a cyclic nucleotide-activated channel from olfactory neurons. Nature 347, 184–187. 10.1038/347184a0 1697649

[B13] Frings S. Lynch J. W. Lindemann B. (1992). Properties of cyclic nucleotide-gated channels mediating olfactory transduction. Activation, selectivity, and blockage. J. Gen. Physiol. 100, 45–67. 10.1085/jgp.100.1.45 1324972 PMC2229120

[B14] González-Maeso J. C. Sealfon S. (2012). Functional selectivity in GPCR heterocomplexes. Mini Rev. Med. Chem. 12, 851–855. 10.2174/138955712800959152 22681249 PMC3549660

[B15] Gurwitz D. Haring R. Heldman E. Fraser C. M. Manor D. Fisher A. (1994). Discrete activation of transduction pathways associated with acetylcholine m1 receptor by several muscarinic ligands. Eur. J. Pharmacol. 267, 21–31. 10.1016/0922-4106(94)90220-8 8206127

[B16] Jones D. T. Reed R. R. (1989). Golf: an olfactory neuron specific-G protein involved in odorant signal transduction. Science 244, 790–795. 10.1126/science.2499043 2499043

[B17] Juilfs D. M. Fülle H. J. Zhao A. Z. Houslay M. D. Garbers D. L. Beavo J. A. (1997). A subset of olfactory neurons that selectively express cGMP-stimulated phosphodiesterase (PDE2) and guanylyl cyclase-D define a unique olfactory signal transduction pathway. Proc. Natl. Acad. Sci. U.S.A. 94, 3388–3395. 10.1073/pnas.94.7.3388 9096404 PMC20380

[B18] Katada S. Hirokawa T. Oka Y. Suwa M. Touhara K. (2005). Structural basis for a broad but selective ligand spectrum of a mouse olfactory receptor: mapping the odorant-binding site. J. Neurosci. 25, 1806–1815. 10.1523/JNEUROSCI.4723-04.2005 15716417 PMC6725943

[B19] Kato A. Katada S. Touhara K. (2008). Amino acids involved in conformational dynamics and G protein coupling of an odorant receptor: targeting gain-of-function mutation. J. Neurochem. 107, 1261–1270. 10.1111/j.1471-4159.2008.05693.x 18803693

[B20] Kenakin T. (1995). Agonist-receptor efficacy. II. Agonist trafficking of receptor signals. Trends Pharmacol. Sci. 16, 232–238. 10.1016/S0165-6147(00)89032-X 7667897

[B21] Klasen K. Corey E. A. Kuck F. Wetzel C. H. Hatt H. Ache B. W. (2010). Odorant-stimulated phosphoinositide signaling in mammalian olfactory receptor neurons. Cell. Signal. 22, 150–157. 10.1016/j.cellsig.2009.09.026 19781634 PMC3581345

[B22] Kleene S. J. Gesteland R. C. (1991). Calcium-activated chloride conductance in frog olfactory cilia. J. Neurosci. 11, 3624–3629. 1941099 10.1523/JNEUROSCI.11-11-03624.1991 PMC6575529

[B23] Knauf P. A. Mann N. A. (1984). Use of niflumic acid to determine the nature of the asymmetry of the human erythrocyte anion exchange system. J. Gen. Physiol. 83, 703–725. 10.1085/jgp.83.5.703 6736917 PMC2215658

[B24] Liberles S. D. Buck L. B. (2006). A second class of chemosensory receptors in the olfactory epithelium. Nature 442, 645–650. 10.1038/nature05066 16878137

[B25] Mombaerts P. (2004). Genes and ligands for odorant, vomeronasal and taste receptors. Nat. Rev. Neurosci. 5, 263–278. 10.1038/nrn1365 15034552

[B26] Offermanns S. (2003). G-proteins as transducers in transmembrane signalling. Prog. Biophys. Mol. Biol. 83, 101–130. 10.1016/S0079-6107(03)00052-X 12865075

[B27] Oka Y. Katada S. Omura M. Suwa M. Yoshihara Y. Touhara K. (2006). Odorant receptor map in the mouse olfactory bulb: *in vivo* sensitivity and specificity of receptor-defined glomeruli. Neuron 52, 857–869. 10.1016/j.neuron.2006.10.019 17145506

[B28] Oka Y. Omura M. Kataoka H. Touhara K. (2004). Olfactory receptor antagonism between odorants. EMBO J. 23, 120–126. 10.1038/sj.emboj.7600032 14685265 PMC1271670

[B29] Pace U. Lancet D. (1986). Olfactory GTP-binding protein: signal-transducing polypeptide of vertebrate chemosensory neurons. Proc. Natl. Acad. Sci. U.S.A. 83, 4947–4951. 10.1073/pnas.83.13.4947 3088569 PMC323861

[B30] Powis G. Seewald M. J. Gratas C. Melder D. Riebow J. Modest E. J. (1992). Selective inhibition of phosphatidylinositol phospholipase C by cytotoxic ether lipid analogues. Cancer Res. 52, 2835–2840. 1316230

[B31] Rasche S. Toetter B. Adler J. Tschapek A. Doerner J. F. Kurtenbach S.. (2010). Tmem16b is specifically expressed in the cilia of olfactory sensory neurons. Chem. Senses 35, 239–245. 10.1093/chemse/bjq007 20100788

[B32] Reisert J. Bauer P. J. Yau K.-W. Frings S. (2003). The Ca-activated Cl channel and its control in rat olfactory receptor neurons. J. Gen. Physiol. 122, 349–363. 10.1085/jgp.200308888 12939394 PMC2234486

[B33] Ritter S. L. Hall R. A. (2009). Fine-tuning of GPCR activity by receptor-interacting proteins. Nat. Rev. Mol. Cell Biol. 10, 819–830. 10.1038/nrm2803 19935667 PMC2825052

[B34] Rose C. R. Ransom B. R. (1997). Regulation of intracellular sodium in cultured rat hippocampal neurones. J. Physiol. 499 (Pt 3), 573–587. 10.1113/jphysiol.1997.sp021951 9130155 PMC1159277

[B35] Scholz P. Kalbe B. Jansen F. Altmueller J. Becker C. Mohrhardt J.. (2016). Transcriptome analysis of murine olfactory sensory neurons during development using single cell RNA-Seq. Chem. Senses. [Epub ahead of print] 10.1093/chemse/bjw003 26839357

[B36] Simasko S. M. (1994). A background sodium conductance is necessary for spontaneous depolarizations in rat pituitary cell line GH3. Am. J. Physiol. 266, C709–C719. 8166234 10.1152/ajpcell.1994.266.3.C709

[B37] Spehr M. Wetzel C. H. Hatt H. Ache B. W. (2002). 3-Phosphoinositides Modulate Cyclic Nucleotide Signaling in Olfactory Receptor Neurons. Neuron 33, 731–739. 10.1016/S0896-6273(02)00610-4 11879650

[B38] Stephan A. B. Shum E. Y. Hirsh S. Cygnar K. D. Reisert J. Zhao H. (2009). ANO2 is the cilial calcium-activated chloride channel that may mediate olfactory amplification. Proc. Natl. Acad. Sci. U.S.A. 106, 11776–11781. 10.1073/pnas.0903304106 19561302 PMC2702256

[B39] Tanaka M. Treloar H. Kalb R. G. Greer C. A. Strittmatter S. M. (1999). G(o) protein-dependent survival of primary accessory olfactory neurons. Proc. Natl. Acad. Sci. U.S.A. 96, 14106–14111. 10.1073/pnas.96.24.14106 10570206 PMC24198

[B40] Ukhanov K. Bobkov Y. Corey E. A. Ache B. W. (2014). Ligand-selective activation of heterologously-expressed mammalian olfactory receptor. Cell Calcium 56, 245–256. 10.1016/j.ceca.2014.07.012 25149566 PMC4188773

[B41] Ukhanov K. Brunert D. Corey E. A. Ache B. W. (2011). Phosphoinositide 3-kinase-dependent antagonism in Mammalian olfactory receptor neurons. J. Neurosci. 31, 273–280. 10.1523/JNEUROSCI.3698-10.2011 21209212 PMC3079265

[B42] Ukhanov K. Corey E. A. Brunert D. Klasen K. Ache B. W. (2010). Inhibitory odorant signaling in Mammalian olfactory receptor neurons. J. Neurophysiol. 103, 1114–1122. 10.1152/jn.00980.2009 20032232 PMC2822701

[B43] Vlahos C. J. Matter W. F. Hui K. Y. Brown R. F. (1994). A specific inhibitor of phosphatidylinositol 3-kinase, 2-(4-morpholinyl)-8-phenyl-4H-1-benzopyran-4-one (LY294002). J. Biol. Chem. 269, 5241–5248. 8106507

[B44] Wong G. T. Gannon K. S. Margolskee R. F. (1996). Transduction of bitter and sweet taste by gustducin. Nature 381, 796–800. 10.1038/381796a0 8657284

[B45] Yu Y. Boyer N. P. Zhang C. (2014). Three structurally similar odorants trigger distinct signaling pathways in a mouse olfactory neuron. Neuroscience 275, 194–210. 10.1016/j.neuroscience.2014.05.063 24929067

[B46] Zocher M. Fung J. J. Kobilka B. K. Müller D. J. (2012). Ligand-specific interactions modulate kinetic, energetic, and mechanical properties of the human β2 adrenergic receptor. Structure 20, 1391–1402. 10.1016/j.str.2012.05.010 22748765 PMC4506644

