# Supplementary figures and images for: Identification of a Novel Gnao-Mediated Alternate Olfactory Signaling Pathway in Murine OSNs
Source: Front Cell Neurosci. 2016 Mar 29;10:63. doi: 10.3389/fncel.2016.00063 (PMC4809895; doi:10.3389/fncel.2016.00063)

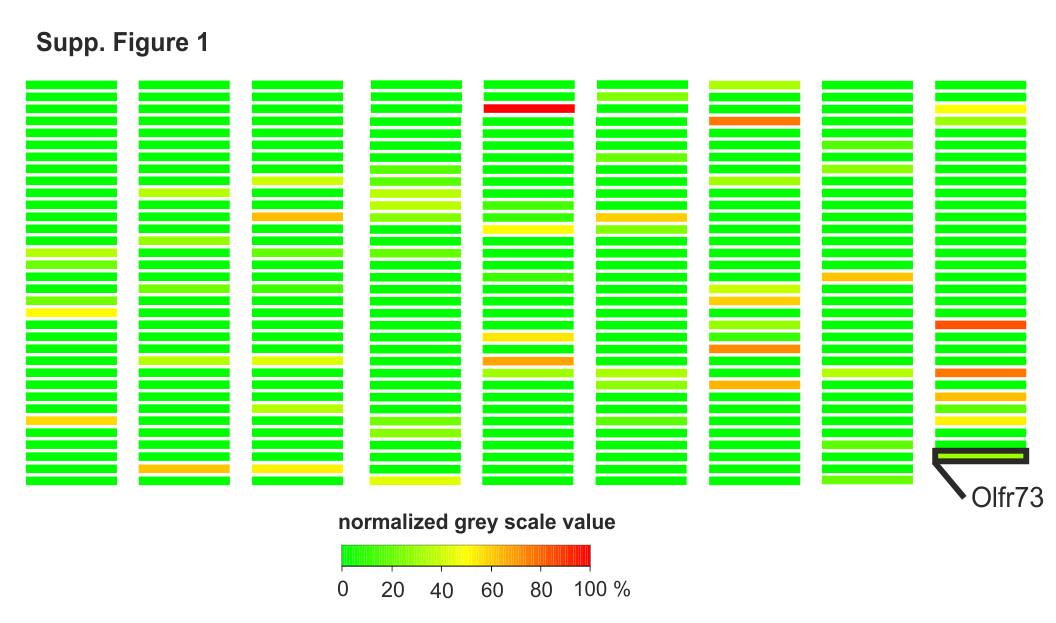

Supplement: Supplementary Figure 1 — Peptide microarrays spotted with 304 OR c-termini and incubated with heterologously expressed Gnao. [file Image1.TIF]
